# An Unusual Association between Neurofibromatosis Type 1 and Diffuse B Cell Lymphoma

**DOI:** 10.1155/2021/5575957

**Published:** 2021-04-13

**Authors:** Anas Bizanti, Ariege Bizanti, Ahmad Al-abdouh, Mohammed Mohammed, Maria Pardi

**Affiliations:** ^1^Saint Agnes Hospital, Baltimore, MA, USA; ^2^University of Central Florida, Orlando, FL, USA

## Abstract

Neurofibromatosis type 1 (NF-1) is known to be associated with increased risk of malignancy by at least fourfold. Malignant lymphomas are rare in adults with NF-1. Hereby, we present a 75-year-old male with NF-1 complaining of weakness, nausea, and vomiting associated with abdominal pain. Three months prior to presentation, he had suffered a motor vehicle accident (MVA) resulting in multiple rib fractures that was seen in chest X-ray. For the following three months, he had intermittent chest pain, but it was attributed to the recent rib fracture. During this admission, the severity of chest pain worsened and the associated vomiting inclined further investigation; including CT imaging and bone biopsy, it was revealed to be a rare case of diffuse B cell lymphoma in a patient with NF-1. However, we believe the recent MVA caused an anchoring bias in making a prompt diagnosis. In addition, the appearance of the neurofibroma, resulted in suboptimal physical examination, and hence, there was a delay in reaching the diagnosis. We will discuss here the presentation of this case, to highlight the rare association and to increase awareness of when encountering a challenging diagnosis.

## 1. Introduction

Neurofibromatosis type 1 (NF-1) or von Recklinghausen disease is known to be associated with increased risk of malignancy by at least fourfold [1]. Most commonly seen are nervous system tumors like optic gliomas, neurofibromyosarcomas, and leukemia [2]. Malignant lymphomas are rare in adults with NF-1 [3–5]. Hereby, we present a rare case of diffuse B cell lymphoma in a patient with NF-1 since childhood.

## 2. Case Presentation

**Figure 1:**
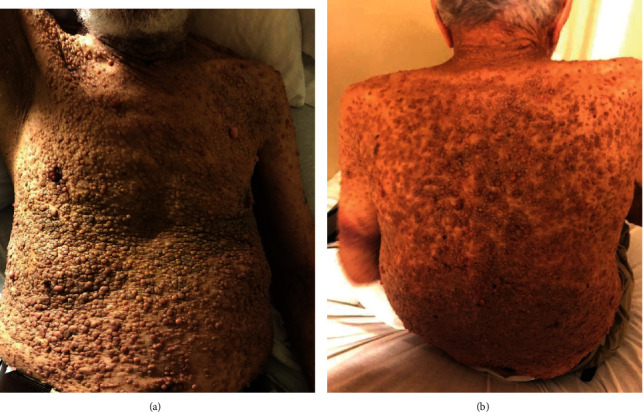
(a, b) The classic neurofibromas of neurofibromatosis type 1 covering the entire surface of the upper extremities and trunk.

**Figure 2:**
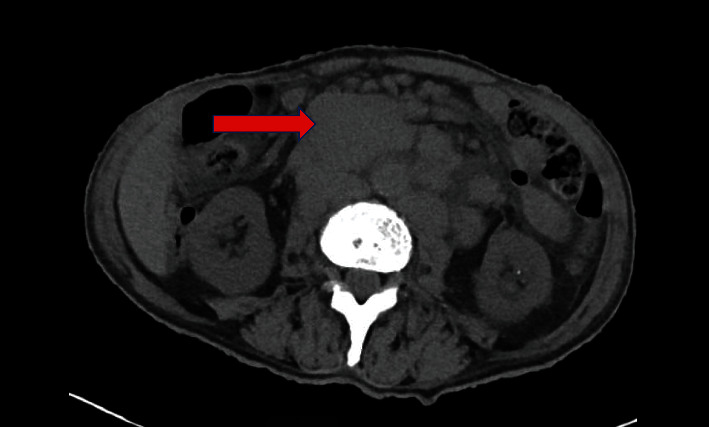
CT of the abdomen, axial view, showing a soft tissue mass anterior to the abdominal aorta and posterior to the body of the pancreas measuring 8 × 6 × 4 cm (red arrow) representing lymphadenopathy from the diffuse B cell lymphoma.

**Figure 3:**
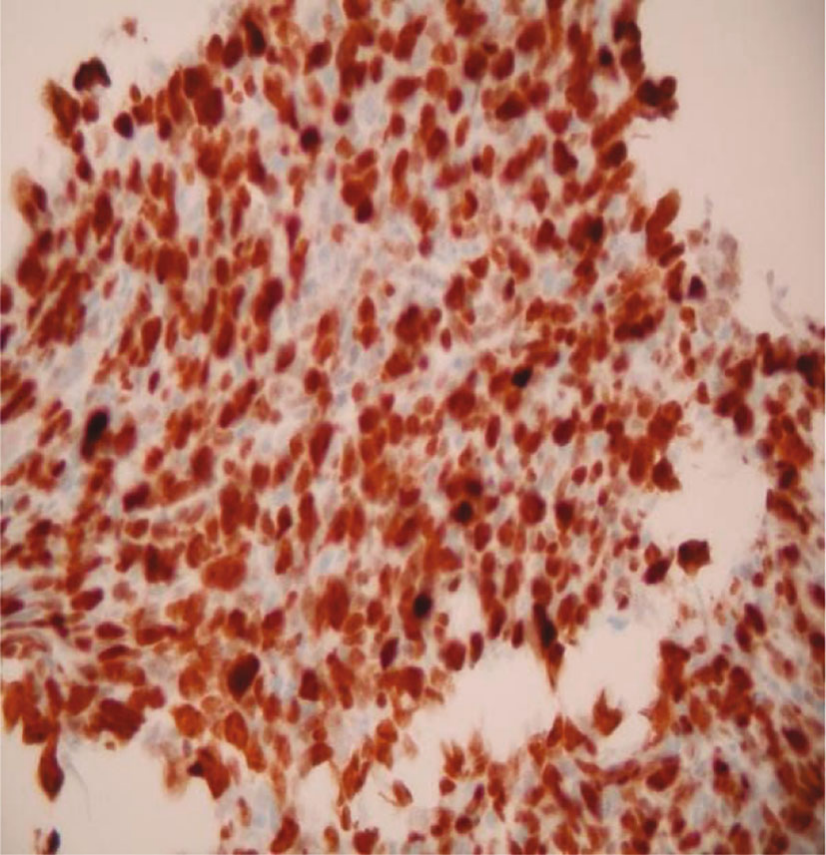
The soft tissue biopsy under H&E stain and high power: no atypia or malignancy noted.

**Figure 4:**
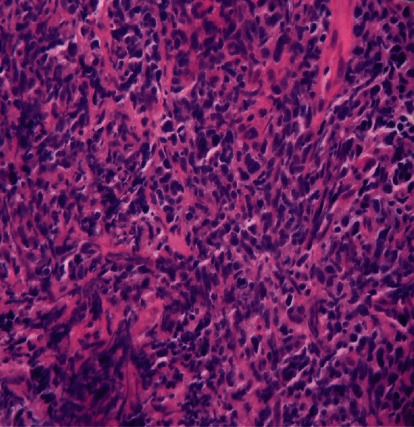
Immunohistochemical stains show that the majority of cells in the specimen are CD20-positive B cells. There is background smaller CD3-positive T-cells. Overall, the findings are most consistent with a diffuse large B cell lymphoma.

A 75-year-old male with NF-1 presented generalized weakness, nausea, and vomiting for three months associated with intermittent epigastric abdominal pain, unspecified weight loss, anorexia, and progressive increase in the number of neurofibromas on the anterior and posterior trunks. His medical history was significant for atrial fibrillation on warfarin and a motor vehicle accident (MVA) that resulted in multiple rib fractures and a grade 1 liver laceration two months prior to presentation. Since the MVA, he has suffered from intermittent abdominal pain that required multiple urgent clinic visits; however, his complaints were attributed to the MVA and dismissed. Physical examination findings included a soft mass in the umbilical area that measured about 8 × 4 cm, was tender to palpation, and had well-defined margins. In addition, he had multiple, small-sized, flesh-colored, dome-shaped fibromas clustered on the trunk, abdomen, and upper extremities (Figure 1). Laboratory investigations demonstrated WBC count 20.0 K/*μ*l with predominant neutrophils, calcium 16.2 mg/dl, parathyroid hormone (PTH) 9.5 pg/ml, PTH-related protein 2.6 pmol/ml, and vitamin D level 15.9 ng/ml. A computed tomography (CT) scan of the abdomen showed a dominant, oval-shaped, soft tissue density in the middle of the upper abdomen, superficial to the abdominal aorta and multiple paraesophageal lymphadenopathy, the largest measuring 8 × 4 × 6 cm (Figure 2). Pathology from a CT-guided biopsy of a retroperitoneal lymph node was consistent with diffuse large B cell lymphoma (DLBCL) (Figures 3 and 4). Bone marrow aspirates did not show atypical cells. Hypercalcemia was managed medically. Subsequently, he was started on treatment with chemotherapy (CHOP). He tolerated the first cycle well with no major side effects and was discharged to a rehabilitation program.

## 3. Discussion

NF-1 is known to be a significant risk factor for malignancy, mainly the peripheral nerve sheath tumors, gliomas, and leukemia. Various studies have estimated the frequency of malignancy in NF-1 patients to be between 5% and 29% [6]. Overall, diffuse B cell lymphoma were reported to be uncommon in association with NF-1. This further contributes to the complexity of clinical heterogeneity associated with NF-1 and the need of a greater understanding of the association between NF-1 and associated malignancies. We performed a literature review through the National library of Medicine (PubMed) searching for case reports of NF-1 associated with lymphoma. Only 29 cases of malignant lymphoma were reported in NF-1 patients [7–9], and only three of them were DLBCL [10–12]. It is worth mentioning that unlike our case, most of the cases reported in previous studies had a history of multiple health conditions that might be confounding variables. Moreover, the American College of Medical Genetics and Genomics (ACMG) has laid a surveillance guideline for adults with NF-1 [13], yet there is no recommendation for imaging nor biomarker surveillance at this point for DLBCL due to it rarity, and thus reporting such cases is a key for physician awareness and to establish surveillance guideline for NF-1 adults' population. Furthermore, the physical presence of the neurofibromas can interfere with the clinician's ability to perform an adequate examination, either due to their anatomical interference during palpation or the unease lesions of this sort might trigger. Another aspect to be highlighted is how the MVA had caused an anchoring bias in the differential diagnosis of abdominal pain which led to a delay in the diagnosis of the lymphoma. Thus, the case presented here should increase clinician's awareness to reach a correct diagnosis when presented with similar case to prevent the patient being denied timely and effective treatment.

## 4. Conclusion

This case emphasizes and enforce the importance of having a high index of suspicion for malignancies during follow-up of patients with a history of NF-1. Finally, physicians should be extremely cautious when examining patients that may impose a challenge on physical examination, in order to avoid biases and delay in diagnosis.

## References

[1] Kallionpaa R. A., Uusitalo E., Leppävirta J., Pöyhönen M., Peltonen S., Peltonen J. (2018). Prevalence of neurofibromatosis type 1 in the Finnish population. *Genetics in Medicine*.

[2] Guillamo J. S., Creange A., Kalifa C. (2003). Prognostic factors of CNS tumours in neurofibromatosis 1 (NF1): a retrospective study of 104 patients. *Brain*.

[3] Jett K., Friedman J. M. (2010). Clinical and genetic aspects of neurofibromatosis 1. *Genetics in Medicine*.

[4] Korf B. R. (2000). Malignancy in neurofibromatosis type 1. *The Oncologist*.

[5] Walker L., Thompson D., Easton D. (2006). A prospective study of neurofibromatosis type 1 cancer incidence in the UK. *British Journal of Cancer*.

[6] Brasfield R. D., Das Gupta T. K. (1972). Von Recklinghausen's disease: a clinicopathological study. *Annals of Surgery*.

[7] Berman B. W., Binder R. A., Cornfield D. B. (1977). Burkitt-lymphoma in a patient with neurofibromatosis and pheochromocytoma. *Jama-Journal of the American Medical Association*.

[8] Cajaiba M. M., Reyes-Mugica M. (2009). Gaucher or pseudo-Gaucher? The challenge of several diseases colliding in a pediatric patient. *Human Pathology*.

[9] Wertelecki W., Iinuma K., Bentley H. P. (1985). Non-neural malignancy complicating neurofibromatosis in two relatives. *Cancer Genetics and Cytogenetics*.

[10] Dohi O., Hatori M., Ichinohasama R., Hosaka M., Hashimoto S., Kokubun S. (2006). Diffuse large B-cell lymphoma arising in a patient with neurofibromatosis type I and in a patient with neurofibromatosis type II. *The Tohoku Journal of Experimental Medicine*.

[11] Kim S. J., Seo J. H., Lee S. W. (2003). A case of non-Hodgkin's lymphoma in a patient with neurofibromatosis type 1. *The Korean Journal of Internal Medicine*.

[12] Lueangarun S., Auewarakul C. U. (2012). Diffuse large B cell lymphoma presenting as Horner's syndrome in a patient diagnosed with neurofibromatosis type 1: a case report and review of the literature. *Journal of Medical Case Reports*.

[13] Stewart D. R., Korf B. R., Nathanson K. L., Stevenson D. A., Yohay K. (2018). Care of adults with neurofibromatosis type 1: a clinical practice resource of the American College of Medical Genetics and Genomics (ACMG). *Genetics in Medicine*.

